# Molecular Characterization of Measles and Rubella Virus Strains from the 2018–2019 Epidemic in Madagascar

**DOI:** 10.3390/pathogens15050514

**Published:** 2026-05-12

**Authors:** Richter Razafindratsimandresy, Jonhson Raharinantoanina, Emmanuel Andrianiriana, Anja Elsam Andrianjakatsilavo, Arvé Ratsimbazafy, Laurence Randrianasolo, Norosoa Harline Razanajatovo, Soa Fy Andriamandimby, Jean Michel Heraud, Vincent Lacoste

**Affiliations:** 1 Unité de Virologie, Institut Pasteur de Madagascar, Antananarivo 101, Madagascar; jonhson@pasteur.mg (J.R.); andrianirianaemmanuel@gmail.com (E.A.); anjaelsam2@gmail.com (A.E.A.); arve@pasteur.mg (A.R.); norosoa@pasteur.mg (N.H.R.); soafy@pasteur.mg (S.F.A.); jean-michel.heraud@pasteur.fr (J.M.H.); vincent.lacoste@pasteur.fr (V.L.); 2 Unité d’Epidémiologie et de Recherche Clinique, Institut Pasteur de Madagascar, Antananarivo 101, Madagascar; laurence@pasteur.mg; 3 Département de Virologie, Institut Pasteur, 75724 Paris Cedex 15, France

**Keywords:** measles, rubella, genotype, Madagascar

## Abstract

Madagascar experienced a severe measles epidemic between September 2018 and mid-2019, resulting in more than 146,000 cases and 1200 deaths, primarily among children under 15 years of age. This epidemic occurred in a context of low vaccination coverage. Prior to this outbreak, no genotyping data were available for the measles virus (MeV) or rubella virus (RuV) in Madagascar. This study aimed to molecularly characterize MeV and RuV strains circulating during the epidemic. A total of 310 biological samples (gingival swabs, urine, and stool) were collected from 288 suspected cases with a mean age of 11.4 years. Viral detection was performed using real-time RT-PCR, followed by conventional RT-PCR and sequencing of the N and H genes for MeV and of the E1 gene for RuV. Co-circulation of the two viruses was observed, with positivity rates of 39.9% (115/288) for MeV and 40.0% (70/175) for RuV. The mean age differed significantly between MeV-positive (12.0 years) and RuV-positive (7.2 years) patients, with 35.1% and 43.8% of cases occurring in children under five years of age, respectively. Phylogenetic analysis showed that all MeV strains belonged to genotype [B3], and they were highly similar to strains circulating globally in 2018–2019, suggesting recent introduction. In contrast, all RuV strains belonged to genotype [2B] and displayed greater genetic diversity, consistent with endemic rubella circulation in a partially vaccinated or unvaccinated population. This study provides the first genotyping data for Madagascar essential for monitoring virus circulation and supporting elimination efforts in the African region.

## 1. Introduction

Measles is a highly contagious infectious disease characterized by high fever, cough, coryza, conjunctivitis, and a distinctive maculopapular rash. It remains a major cause of morbidity and mortality worldwide. The causative agent, measles virus (MeV), is an enveloped, non-segmented negative-sense single-stranded RNA virus. MeV is a member of the genus *Morbillivirus* within the family *Paramyxoviridae*. Its genome contains six genes encoding the nucleoprotein (N), phosphoprotein (P), matrix (M), fusion (F), hemagglutinin (H), and polymerase (L) proteins. MeV strains are divided into eight clades (A–H) and 24 genotypes (A, B1 to B3, C1 and C2, D1 to D11, E, F, G1 to G3, H1, and H2) [1,2].

Rubella, also characterized by a maculopapular rash and fever, is generally a mild disease primarily affecting children. Complications of rubella include arthritis and, more rarely, encephalitis, but infection with Rubella virus (RuV) is also associated with severe birth defects when women are infected early in pregnancy, with long-term health consequences for children born with congenital rubella syndrome (CRS) [3,4]. RuV belongs to the genus *Rubivirus* within the family *Matonaviridae* [5]. RuV is an enveloped virus with a non-segmented, positive-sense single-stranded RNA genome. Its genome encodes two non-structural proteins (P90 and P150) and three structural proteins (C, E1, and E2) [6]. On the basis of E1 gene sequences, RuV strains are divided into two clades. Clade 1 is divided into 10 genotypes (1a and 1B to 1J), and clade 2 into three (2A to 2C). Of these, four genotypes, namely 1E, 1G, 1J, and 2B, are reported to circulate commonly in different regions of the world [7].

Both measles and rubella are vaccine-preventable diseases. With measles and rubella elimination efforts underway in many countries, the distribution of genotypes may change, with some previously common genotypes becoming less frequent as vaccination coverage increases. To effectively monitor the interruption of endemic transmission of MeV and RuV and track changes in genotype distribution, viral sequence characterization combined with case investigations is therefore essential [8,9].

In Madagascar, the Expanded Program on Immunization (EPI) has been in place since 1976 and includes measles-containing vaccines (MCVs) in its vaccination calendar. Until 2020, a single dose of MCV was administered at 9 months of age. MCV2, scheduled between 15 and 18 months of age, was introduced in October 2020. Following mass vaccination campaigns in September and October 2004, national surveillance of suspected measles cases was established. Since then, the National Reference Laboratory (NRL) for measles and rubella, hosted by the Virology unit at the Institut Pasteur de Madagascar, has been responsible for serological diagnosis using ELISA. From late 2004 to August 2018, the NRL received and tested 7072 sera, of which 39 (0.5%) and 1762 (24.9%) were IgM-positive for MeV and RuV, respectively. Starting in epidemiological week 35 (September) of 2018, Madagascar experienced a massive measles epidemic, which was officially declared nationwide in October 2018. By May 2019, the country had reported 146,277 measles cases, including 1394 laboratory-confirmed cases and 144,883 cases confirmed by epidemiological link [10]. The number of deaths reached over 1200 people, predominantly children under 15 years of age. The epidemic continued through the first half of 2019 but began to subside around mid-2019 following extensive vaccination campaigns. During the emergency response, about 7.2 million children aged six months to nine years were vaccinated [10]. The outbreak was largely controlled by September 2019.

Prior to this epidemic, Madagascar had one of the lowest measles vaccination coverage rates globally. According to the WHO–UNICEF estimates of national coverage (WUENIC) data, the coverage of MCV1 in Madagascar was estimated to be between 55% and 81% from 2002 to 2018, while the administrative coverage was between 61% and 98% (https://worldhealthorg.shinyapps.io/wuenic-trends/, accessed on 23 May 2025). The low coverage was likely related to vaccine hesitancy and limited access to vaccination services in certain regions of the country. This Malagasy outbreak was one of the largest during the global measles resurgence of 2018–2019, which affected all regions of the world, with other major hotspots in Ukraine, India, the Philippines, and parts of Europe and the USA.

During the epidemic, the NRL implemented molecular biology techniques to diagnose and genotype both viruses. In parallel, additional biological samples (gingival, urine, and stool) were collected from suspected measles patients for molecular confirmation of suspected cases and genetic characterization of viruses from confirmed cases. This study aimed to molecularly characterize the MeV and RuV strains that circulated in Madagascar during the 2018–2019 epidemic.

## 2. Materials and Methods

### 2.1. Ethics Statement

For the patient samples included in the study, the participant or a parent or legal guardian provided written informed consent. Ethical approval was obtained from the Ethics Committee for Biomedical Research of the Ministry of Public Health of Madagascar (Comité d’Ethique de la Recherche Biomédicale auprès du Ministère de la Santé Publique de Madagascar # 96/MSANP/CERBM) issued on 28 August 2018.

### 2.2. Sample Collection

For patients suspected of having measles, one to three types of samples were collected. Samples were collected within the first four days after rash onset. Gingival specimens were swabbed and preserved into viral transport medium (VTM). Urine samples were collected in sterile tubes and stool samples by rectal swabbing. After collection, all samples were sent to the NRL at 4 °C and either processed immediately upon arrival or stored at −80 °C until analysis. A total of 310 specimens, consisting of 288 gingival swabs, 13 stool, and 9 urine samples, were collected from 288 patients (Table 1).

### 2.3. Molecular Screening for MeV and RuV

Viral RNA extraction from 140 µL of clinical specimens was performed using the QIAamp^®^ Viral RNA mini kit (Qiagen, Hilden, Germany) according to the manufacturer’s instructions. RNA was eluted in a final volume of 65 µL and stored at −80 °C. Real-time RT-PCR assays were performed for the detection of measles and rubella viruses on an ABI 7500 Real-Time PCR system (Applied Biosystems, Foster City, CA, USA), following CDC protocols. Primers, probes, and control RNAs were provided by CDC as kits. Primers and probe for MeV were described by Hummel et al., and those for RuV by Schulz et al., respectively [11,12]. In addition, RNA presence and integrity were confirmed using primers specific to the human RNase P gene [11,12,13]. For MeV, samples with a C_t_ value below 38 were considered positive and used for genotyping. All negative specimens for MeV were then tested for RuV. Those with a C_t_ value below 40 were considered positive and further used for genotyping.

### 2.4. Genotyping of MeV and RuV

All samples positive for MeV and RuV by real-time RT-PCR were subsequently tested by conventional RT-PCR for genotyping. Genetic characterization was based on sequence analysis of the N and H genes for MeV and the E1 gene for RuV, following previously published protocols [14,15,16,17,18]. Positive controls (RNA provided by CDC) and negative controls (RNase-free water) were included in each run. PCR products were visualized by agarose gel electrophoresis prior to sequencing.

### 2.5. Phylogenetic Analysis

Amplification products were sent for sequencing to GENEWIZ Europe (Leipzig, Germany). Raw sequences were analyzed and edited using CLC Genome Workbench 8.1. (Qiagen, Aarhus A/S, Aarhus, Denmark). Then, for each gene, a multiple-sequence alignment was built using the ClustalW program, using the obtained sequences and other closely related sequences via BLAST + 2.16.0 (blast.ncbi.nlm.nih.gov/Blast.cgi), as well as WHO reference sequences and sublineage reference sequences retrieved from the GenBank, MeaNS, and RubeNS databases. The sequences were translated into amino acids, and both nucleotide and amino acid sequences were checked for irregularities. Pairwise sequence identity (at the nucleotide and amino acid levels) of the different coding sequences was calculated with MEGA version 7 (www.megasoftware.net) using uncorrected p-distances [19]. Phylogenetic trees were inferred from the aligned nucleotide sequences by using a maximum likelihood phylogenetic approach, applying the best-fitted model of nucleotide substitution for our datasets, as determined using MEGA v7 under corrected Akaike information criteria (AICc). Five hundred bootstrap replicates were generated.

### 2.6. Statistical Analyses

Statistical analyses were performed using R software 4.4.2 version (http://www.R-project.org/). Univariable logistic regression was performed to evaluate the association between age groups and viral positivity. Statistical significance was assessed using *p*-values derived from the Wald test.

### 2.7. Accession Numbers

Sequences reported in this paper were named according to WHO recommendations and were deposited in the GenBank database under accession numbers PV648754 to PV648867, PV659707 to 659779, and PV648868 to PV648912 for the N, H, and E1 sequences, respectively. In the MeaNS database, they are available under ID numbers (MeaNS2 Seq. id.) 172788 to 172803; 172805 to 172807; 172809; 172811 to 172834; and 172844 to 172849. In the RubeNS database, the sequences are available under ID numbers (RubeNS2 Seq. id.) 16648 to 16678.

## 3. Results

A total of 310 samples were collected from 288 suspected measles patients: three types of samples (gingival, stool and urine) were collected for nine patients, two types of samples (gingival and stool) for four patients and only gingival samples for 275 patients (Table 1). The male-to-female ratio (M:F) was 0.79 (127/161), with a mean age of 11.4 years (range: 0–65 years).

All samples were tested for MeV by quantitative RT-PCR. The positivity rate was 39.9% with 115 of 288 patients testing positive for MeV (Table 1 and Table 2). The mean age of the positive patients was 12.0 years (range: 0.1–65 years). The overall sample-based detection rate of MeV was 40.3% (125/310) with 39.9% of gingival samples (115/288), 61.5% of stool (8/13), and 22.2% of urine samples (*n* = 2/9) being positive (Table 2).

Of the 125 samples positive for MeV by quantitative RT-PCR, 111 were positive for the N gene using conventional RT-PCR: 101 gingival samples (87.8%), eight stool samples (100%), and two urine samples (100%). In addition, 65 samples were positive for both H1 and H2 gene fragments, including 57 gingival samples (49.6%), seven stool samples (87.5%), and one urine sample (50%) (Table S1). The 14 samples negative for both genes had C_t_ values greater than 33.9 in the real-time assay. Samples (n = 11) with discrepancies in H1 and H2 amplifications had C_t_ values ranging from 21.6 to 24.5 in the real-time assay.

**Figure 1:**
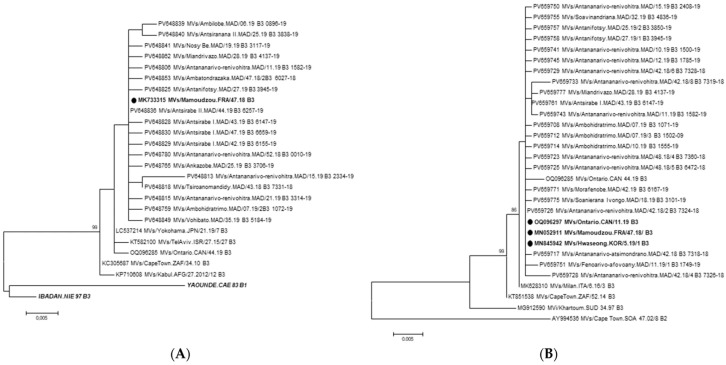
Phylogenetic analysis of the N gene (450 bp) (**A**) and H gene (936 bp) (**B**) sequences of MeV. The scale bar indicates the number of nucleotide substitutions per site. Isolates with solid black circles represent the closest available sequences, and those in bold and italic correspond to reference viruses. Only bootstrap values > 75% are indicated.

For the N gene, 100 sequences of 450 bp (nucleotide positions 1126–1575 according to Y-14 strain, accession number U01998) were obtained from the 111 amplicons sent for sequencing. The remaining sequences were partial and were excluded from further analyses. These sequences exhibited 98.9% to 100% nucleotide identity and 98.7% to 100% amino acid identity across the 450 bp (150 aa) sequences. They showed 99.1% to 100% nucleotide identity with the MVs/Mamoudzou.FRA/47.18 [B3] strain detected in Mayotte (Comoros Islands, Indian Ocean) in 2018. In addition, 47 sequences of 936 bp of the H gene (nucleotide positions 510–1445 according to the MVs/Milan.ITA/3.16/2 [B3] strain, accession number MK628300) were obtained from the 65 samples from which the H1 + H2 fragments were amplified. They displayed 99.7% to 100% nucleotide identity and 97.1% to 100% amino acid identity among themselves over the 936 bp (312 aa) sequences. They shared 99.8% to 100% nucleotide identity with MVs/Mamoudzou.FRA/47.18 [B3], MVs/Hwaseong.KOR/5.19/1 [B3], and MVs/Ontario.CAN/11.19 [B3] strains, identified in 2018 and 2019. Phylogenetic analyses were performed on the nucleotide sequences of the N and H genes of MeV (Figure 1). To construct the trees, and for clarity, given that many sequences had 100% nucleotide identity between them, only one sequence from each was used (Figure 1). Consequently, 18 and 22 Malagasy sequences of the N and H genes were included, respectively. All MeV strains detected during this outbreak belonged to the [B3] genotype.

In another hand, search for RuV was carried out in 175 patients: 173 who tested negative for MeV (including five patients from whom three types of samples were collected and 168 from whom only a gingival sample was collected), and the two from whom three types of samples were collected but whose urine samples were negative for MeV (Table 1). The M:F ratio was 0.84 (80/95), and the mean age was 10.9 years (range: 0–61 years). The RuV positivity rate was 40.0% (70/175) (Table 1 and Table 2). The mean age of the positive patients was 7.2 years (range: 0.6–33.8 years). The overall sample-based positivity rate was 40.0% (74/185), including 39.9% of gingival samples (69/173), 60% of stool (3/5), and 28.6% of urine samples (2/7) being positive (Table 2).

**Figure 2:**
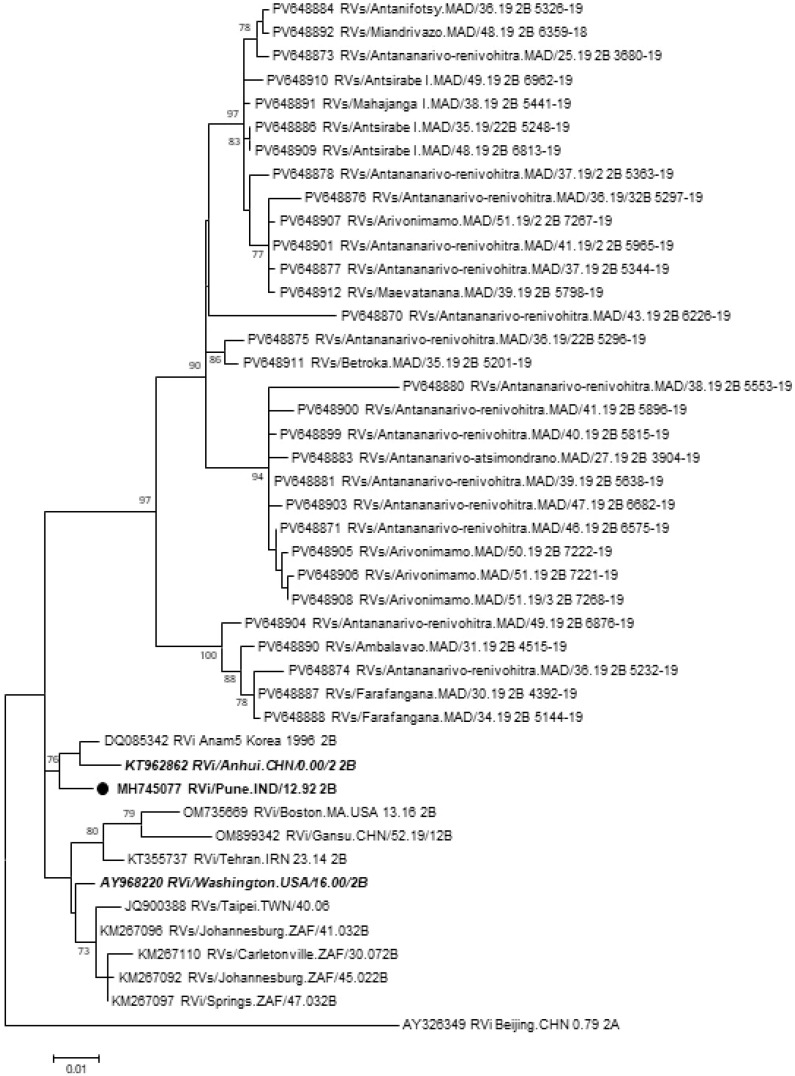
Phylogenetic analysis of E1 gene sequences (739 bp) of RuV. The scale bar indicates the number of nucleotide substitutions per site. Sequences in bold and italic correspond to reference viruses. Bootstrap values are indicated when >70%.

Of the 74 samples positive for RuV by quantitative RT-PCR, 47 (63.5%) were positive for the E1 gene by conventional RT-PCR, including 44 gingival samples (63.8%), one stool sample (33.3%), and two urine samples (100%) (Table S2). The 27 negative samples had C_t_ values greater than 31.4 in the real-time assay. Forty-three E1 gene sequences were obtained. Sequences of 739 bp in length (nucleotide positions 8731 to 9469 according to the RVi/Seattle.WA.USA/16.00 [2B] strain, accession number JN635293) were obtained for 31 of the 43 RT-PCR products sent for sequencing. They exhibited between themselves 94.3% to 99.9% nucleotide identity and 94.3% to 100% amino acid identity over the 739 bp (246 aa) sequences. They were most closely related to the RVi/Pune.IND/12.92 [2B] strain, showing 94.7% to 96.3% nucleotide identity. BLAST analyses confirmed that all sequences belonged to genotype [2B]. Of note, all Malagasy strains possessed signature mutations at positions G8805C, C8904T, and T/C8958A (nucleotide positions relative to JN635293), compared to other sequences of genotype [2B]. These three polymorphic sites were nevertheless synonymous substitutions. Phylogenetic analysis demonstrated that all Malagasy sequences formed a single, well-supported monophyletic clade (bootstrap value = 97), distinct from RuV sequences from other countries (Figure 2). In addition, they displayed substantial genetic diversity. Within this monophyletic clade, they branched into several distinct well-supported monophyletic sub-clades in which some Malagasy sequences are as genetically distant from each other as some international strains are from one another.

## 4. Discussion

This study reveals the co-circulation of MeV (genotype [B3]) and RuV (genotype [2B]) in Madagascar during the 2018–2019 epidemic, with positivity rates of approximately 40% among the tested specimens for each virus.

The specimens analyzed in this study were collected through the national laboratory surveillance system for case confirmation and were not intended to constitute a statistically random sample of all measles cases reported nationally during the outbreak. Nevertheless, they showed broad geographic coverage, with samples originating from all six provinces of Madagascar (22 out of 23 regions), and the demographic characteristics were broadly consistent with national surveillance data [10]. The age distribution of tested suspected cases was broadly consistent with national surveillance data, with a median age of 6.5 years, compared with 6 years nationally, and 73.3% of tested cases aged less than 15 years versus 77% nationally. Sex distribution was also broadly comparable, although females were slightly overrepresented in the tested subset. Vaccination history was insufficiently documented for robust comparison. Overall, these findings support that the molecular dataset reasonably reflected the main epidemiological patterns of the national outbreak and was suitable for virological analysis.

Among tested suspected cases, 33.9% (39/115) of MeV-positive patients and 45.7% (32/70) of RuV-positive patients were children under 5 years of age (Table 3). The substantial burden of both viruses among suspected cases, together with their distinct age distributions (average 12.0 years for MeV vs. 7.2 years for RuV), confirms gaps in vaccination coverage and/or waning immunity in the population. The higher measles positivity observed among individuals aged 10–14 years may also reflect the accumulation of susceptible cohorts resulting from historical gaps in routine immunization and incomplete catch-up vaccination. This interpretation is consistent with previous work in Madagascar showing that major measles outbreaks can occur when immunity deficits build up across successive birth cohorts [20,21].

The positivity rates varied across sample types, ranging from 22.2% to 61.5% for MeV and from 28.6% to 60% for RuV, with stool samples showing the highest rates for both viruses. Gingival specimens yielded similar positivity rates of approximately 40% for both viruses. However, the small number of urine and stool samples limits interpretations regarding the optimal specimen type. In any case, although the search for MeV in stool is uncommon, this study demonstrates the value of this sample type for the molecular diagnosis of measles and rubella, as has already been shown for oral/gingival and urine samples by numerous studies worldwide [22,23,24,25,26,27,28,29,30,31,32].

Molecular characterization also provides insights into transmission dynamics. Malagasy MeV strains were highly similar to strains that circulated in Mayotte, South Korea, and Canada in 2018–2019, suggesting recent introduction or linkage to broader circulation patterns of a globally circulating [B3] genotype. In contrast, Malagasy RuV strains displayed greater within-country genetic diversity and formed a distinct monophyletic clade, suggesting local circulation over time. While the substantial genetic diversity within Malagasy sequences may indicate long-term endemic persistence with multiple co-circulating lineages, the absence of international strains clustering with Malagasy ones suggests that recent introductions have either not occurred or have not established persistent transmission chains. These findings indicate that rubella elimination in Madagascar is achievable but would require sustained high vaccination coverage and strong surveillance across all regions of the country.

## 5. Conclusions

Genotyping of MeV and RuV is a key component of measles and rubella surveillance and elimination strategies. This study provides the first molecular data on circulating MeV and RuV strains in Madagascar, and it improves our understanding of virus circulation in the African region. Sustained molecular monitoring will be essential to evaluate the impact of vaccination programs, document interruption of endemic transmission, detect reintroduction rapidly, and support national and global measles and rubella elimination goals.

## Figures and Tables

**Table 1:** Detection of measles virus (MeV) and rubella virus (RuV) by patient and specimen types.

	MeV				RuV			
	Individuals			Total of Specimen	Individuals			Total of Specimen
Specimen Types	Number	Positive	Negative		Number	Positive	Negative	
Gingival, Stool, Urine	7	2	5	21	5	3 †	2	15
Gingival+, Stool+, Urine− *	2	2	0	6	2 ^#^	0	2	2
Gingival, Stool	4	4	0	8	0	0	0	0
Gingival	275	107	168	275	168	67	101	168
Total	288	115	173	310	175 ^!^	70	105	185

* Indicates the number of patients with three specimen types for whom gingival and stool samples were positive, while the urine sample was negative for MeV. ^#^ Refers to the two urine samples that were negative for MeV. ^!^ A total of 175 individuals were tested for RuV, rather than 173 (i.e., those negative for MeV), given that two individuals for which urine samples were negative for MeV were included. † All three specimen types were positive for RuV in one patient, while in the other two patients, either gingival and stool samples or urine and stool samples were positive.

**Table 2:** Frequency of measles virus (MeV) and rubella virus (RuV) detection by specimen types using real-time RT-PCR.

Specimen Types	MeV Positive *n* (%)	Total Tested	RuV Positive *n* (%)	Total Tested
Gingival	115 (39.9)	288	69 (39.9)	173
Stool	8 (61.5)	13	3 (60.0)	5
Urine	2 (22.2))	9	2 (28.6)	7
Total	125 (40.3)	310	74 (40.0 )	185

**Table 3:** Detection of measles virus (MeV) and rubella virus (RuV) by age groups.

**Age Category**	**MeV-Positive** ***n*** **(%)**	**Total Tested**	***p-*** Value *	**RuV-Positive** ***n*** **(%)**	**Total Tested**	***p*** -Value *
[0–5]	39 (35.1)	111		32 (43.8)	73	
[5–10]	18 (28.1)	64	0.85	26 (55.3)	47	0.31
[10–15]	22 (61.1)	36	0.001	6 (42.9)	14	0.94
≥15 years	36 (47.4)	76	0.014	5 (12.5)	40	0.001
Unknown	0 (0.0)	1		1 (100)	1	
	115 (39.9)	288		70 (40.0)	175	

* *p*-values correspond to the comparison for the 10 to <15 years age group (reference category) versus every age group for both MeV and RuV.

## Data Availability

The original contributions presented in this study are included Material and methods/Accession number section. Further inquiries can be directed to the corresponding author.

## Supplementary Materials

The following supporting information can be downloaded at https://www.mdpi.com/article/10.3390/pathogens15050514/s1, Table S1: Genotyping results per gene (and fragment) and specimen type for MeV; Table S2: Genotyping results per specimen type for RuV.
