# Supplementary material for: Molecular Characterization of Measles and Rubella Virus Strains from the 2018–2019 Epidemic in Madagascar
Source: Pathogens. 2026 May 12;15(5):514. doi: 10.3390/pathogens15050514 (PMC13209841; doi:10.3390/pathogens15050514)
Supplement: Supplementary file 1 [file pathogens-15-00514-s001.zip › pathogens-4238558-supplementary.pdf]

Table S1. Genotyping results per gene (and fragment) and specimen type for MeV

	Fragment	Gingival	Stool	Urine	Total
**Amplification**	N pos., H1 pos., H2 pos.*	57	7	1	**65**
	N pos., H1 neg., H2 neg.	34	0	1	**35**
	N pos., H1 neg., H2 pos.	5	1	0	**6**
	N pos., H1 pos., H2 neg.	5	0	0	**5**
	N neg., H1 neg., H2 neg.	14	0	0	**14**
	**Total**	**115**	**8**	**2**	**125**
**Sequencing**	Gene N	101	8	2	**111**
	Gene H#	57	7	1	**65**

* pos. stands for positive and neg. for negative

# numbers reported here correspond to the number of samples from which H1+H2 fragments have been obtained and sequenced

Table S2. Genotyping results per specimen type for RuV

	Gingival	Stool	Urine	Total
**Amplification**	44	1	2	**47**
**Sequencing**	42	0	1	**43**
